# Robotic right colectomy with complete mesocolic excision, central vascular ligation and hand-sewn intracorporeal anastomosis: feasibility, safety, and learning curve analysis

**DOI:** 10.3389/fsurg.2025.1740276

**Published:** 2026-01-14

**Authors:** Zsolt Madarasz, Krysztof Nowakowski, Michael Leitz, Bogdan-Cornel Sturzu, Anas Baltamar, Kira Baginski, Annika Hoyer, Jens Hoeppner, Fabian Nimczewski, Miljana Vladimirov

**Affiliations:** 1 Department of Surgery, Bielefeld University, Medical School and University Medical Center OWL—Campus Hospital Lippe, Detmold, Germany; 2 Biostatistics and Medical Biometry, Medical School OWL, Bielefeld University, Bielefeld, Germany

**Keywords:** complete mesocolic excision, CUSUM, intracorporeal anastomosis, learning curve, right colectomy, robotic surgery

## Abstract

**Background:**

Robotic right colectomy (RRC) with complete mesocolic excision (CME) and central vascular ligation (CVL) has become a standard oncologic approach for right-sided colon cancer. However, evidence regarding hand-sewn intracorporeal anastomosis (ICA) and its associated learning curve remains limited.

**Methods:**

This single-center retrospective study analyzes a series of consecutive patients with histologically confirmed right-sided colon adenocarcinoma who underwent fully robotic RRC with CME, CVL, and hand-sewn ICA. Perioperative outcomes, pathological results, and the learning curves of three colorectal surgeons were evaluated using cumulative sum (CUSUM) and risk-adjusted CUSUM (RA-CUSUM) methods.

**Results:**

Overall, 71 patients were treated by RRC from April 2021 through December 2024. All surgical procedures were completed robotically. The median operative time was 165 min (Q1–Q3: 147–192). Major complications (Clavien–Dindo ≥ IIIb) occurred in 9.9% of cases, with an anastomotic leak rate of 5.6%. Mean lymph-node yield was 29.6 ± 11.2, and R0 resection was achieved in 98.6%. The CUSUM learning curves for the three surgeons revealed a comparable trend, starting with a rise during the learning phase and followed by a decline reflecting increased efficiency. The learning curve plateau was reached after approximately 16 cases for each surgeon.

**Conclusion:**

RRC with CME, CVL, and hand-sewn ICA is feasible, safe, and oncologically effective. Proficiency is typically achieved after 15–20 cases, supporting its role as a reproducible and teachable procedure in structured robotic colorectal programs.

## Introduction

Minimally invasive surgery (MIS) has become the standard approach for many colorectal procedures due to its association with reduced postoperative pain, faster recovery, and lower morbidity compared to open surgery (1). Although laparoscopy has long dominated MIS, robotic surgery is increasingly applied for complex procedures such as right hemicolectomy, where three-dimensional vision and articulated instruments offer distinct ergonomic advantages (2, 3).

One such complex technique is the Complete Mesocolic Excision (CME) with Central Vascular Ligation (CVL), which aims to optimize oncologic outcomes by preserving embryological planes and increasing lymph node harvest (1, 4–6). Robotic systems facilitate CME by providing stable three-dimensional visualization and superior precision during central vessel dissection. Several studies have confirmed the feasibility and oncologic adequacy of robotic CME compared with the laparoscopic approach, with similar or improved lymph-node yields, lower conversion rates, and shorter hospital stay (7–9).

Another evolving aspect of right hemicolectomy is the technique of ileocolic anastomosis. While extracorporeal anastomosis (ECA) remains common, increasing evidence supports the use of intracorporeal anastomosis (ICA) due to its association with better gastrointestinal recovery, fewer wound complications, and reduced incisional hernias. Meta-analyses and comparative studies have confirmed these findings across both laparoscopic and robotic platforms (10–14).

In particular, the hand-sewn ICA technique offers certain theoretical advantages over stapled anastomoses, including greater flexibility in bowel orientation, improved vascular preservation, and lower cost (15). A multicenter prospective cohort study demonstrated that both stapled and hand-sewn ICA in Robotic right colectomy (RRC) is safe and results in comparable anastomotic leak rates (3.3% vs. 3.8%) and morbidity. The robotic system's enhanced suturing capabilities make the hand-sewn approach more accessible and reproducible, particularly for experienced laparoscopic surgeons (7, 15).

Despite continuous technological advances, the learning curve for robotic right RRC remains a major factor limiting its widespread adoption. Analyses using the CUSUM method have shown that surgical proficiency appears achievable after approximately 25–35 procedures, with marked reductions in operative time, intraoperative blood loss, and conversion rates once this threshold is reached (2, 3, 16).

Right colectomy is widely regarded as an ideal index procedure for robotic training due to its reproducible anatomy, standardized dissection planes, and multi-quadrant workflow involving CME and CVL (17). Early series have also confirmed the safety and oncologic adequacy of this approach even during the initial learning phase (15).

However, evidence regarding the integration of hand-sewn ICA within robotic CME workflows, and the associated learning process, remains limited—particularly in multi-surgeon, real-world institutional settings. Existing literature has focused predominantly on stapled ICA, leaving a notable gap concerning the perioperative performance, safety, and reproducibility of the hand-sewn technique in robotic right colectomy.

The present study therefore aimed to evaluate the feasibility, safety, and oncologic outcomes of RRC with CME, CVL, and hand-sewn ICA. Additionally, it sought to characterize the learning curves of three colorectal surgeons using both CUSUM and RA-CUSUM analyses. By standardizing the SMV-first approach and hand-sewn ICA across all cases, this study provides a comprehensive multi-surgeon assessment of a technique for which contemporary evidence remains scarce.

## Materials and methods

### Study design and population

This retrospective single-center study was conducted at the Department of Surgery, University Hospital OWL, Campus Lippe, Bielefeld University, Germany. Data were extracted from a prospectively maintained institutional colorectal cancer database covering the period from April 2021 to December 2024. To ensure methodological consistency cases requiring conversion to open surgery and cases with different anastomotic techniques were excluded.

The primary objective was to evaluate the feasibility and safety of RRC with CME, CVL, and hand-sewn ICA. The secondary objective was to analyze the learning curves of three colorectal surgeons using CUSUM and RA-CUSUM methods.

All procedures were performed by three board-certified colorectal surgeons working in an accredited colorectal cancer center. All surgeons had substantial prior experience in advanced minimally invasive colorectal surgery, including both laparoscopic and robotic resections, with the majority of their robotic experience derived from left-sided colon and rectal procedures.

Robotic CME was offered to all patients with right-sided colon cancer, including tumors of the caecum, ascending colon, hepatic flexure, and proximal transverse colon.

Inclusion criteria

1. Adults aged ≥18 years
2. Histologically confirmed right-sided colon adenocarcinoma
3. Elective, fully robotic right hemicolectomy with CME and CVL
4. Reconstruction using a hand-sewn intracorporeal anastomosis

Exclusion criteria

1. Conversion to open surgery
2. Use of a stapled intracorporeal anastomosis
3. Emergency procedures
4. Synchronous major abdominal procedures

Following application of these criteria, 71 patients were included in the final analysis.

### Preoperative evaluation and preparation

Preoperative staging included contrast-enhanced computed tomography (CT) of the abdomen, pelvis, and thorax to evaluate metastases. Tumors were tattooed preoperatively with ink to facilitate intraoperative localization. Patients were admitted one day before surgery and received both mechanical and oral bowel preparation according to institutional protocol. Prophylactic single-shot intravenous antibiotics were administered at induction of general anesthesia.

### Surgical technique

All procedures were performed using the Da Vinci X® robotic platform (Intuitive Surgical, Sunnyvale, CA, USA). The technique followed the “superior mesenteric vein (SMV)-first” approach as originally described and later validated as safe and feasible in subsequent studies **(**5, 18).

Patients were placed in a modified Lloyd-Davis position and secured on an anti-slip mattress. The table was tilted 10° Trendelenburg and 15° left tilts to facilitate medial retraction of the small bowel. A linear, oblique port configuration was standard: four 8-mm robotic trocars arranged diagonally, each spaced 7–8 cm apart, and one 12-mm assistant port in the left lower quadrant. The robot was docked from the patient's right shoulder (Supplementary Figure S1).

After pneumoperitoneum induction (12–15 mm Hg CO₂) using a Veres needle at Palmer's point, the procedure began with a peritoneal incision over the superior mesenteric vein (SMV) pedicle to expose its anterior surface. Lymphatic and adipose tissue were carefully removed to skeletonize the vein. The ileocolic vein (ICV) was divided between clips at its confluence with the SMV, followed by ligation of the ileocolic artery (ICA) at the right border of the SMV. Dissection then proceeded cranially along the SMV to identify the middle colic artery (MCA), and its right branch was divided between clips to complete a standard right colectomy (Supplementary Figure S2).

Henle's trunk was exposed and dissected with preservation of the gastroepiploic and pancreaticoduodenal veins. Medial-to-lateral dissection was carried out to separate the mesocolon from Gerota's fascia, the duodenum, and the pancreas. The greater omentum was divided along the midline to enter the lesser sac. The gastrocolic ligament was divided external to the gastroepiploic arcade, and mobilization was extended to the hepatic flexure.

After mesenteric division with a robotic vessel sealer, perfusion of bowel ends was assessed using indocyanine-green (ICG) fluorescence. The terminal ileum and transverse colon were transected with a SIGNIA™ Tri-Staple™ stapler (Covidien, USA).

An isoperistaltic, side-to-side intracorporeal anastomosis was hand-sewn using 3-0 V-Loc™ barbed sutures in a single seromuscular layer: posterior wall first, then anterior wall (Supplementary Figures S3–S6). A hand-sewn ICA was selected because it is cost-effective, supports the development of suturing proficiency within the robotic training program, and, based on current evidence, has not been shown to be inferior to stapled anastomoses in terms of safety or clinical outcomes. The specimen was extracted through the Pfannenstiel incision protected with an Alexis® wound retractor; fascia and skin were closed with 0 Vicryl® and 3-0 Caprosyn®.

### Variables and statistical analysis

Demographic, intraoperative, and postoperative data were collected prospectively. Pathologic parameters included T-, N-, and M-staging, grading, R0 resection status, and CME quality [Benz classification (19)]. Complications were classified according to the Clavien–Dindo system (20), and 30-day mortality was recorded.

Patient characteristics, perioperative, postoperative, and histopathological outcomes were first analysed descriptively. Continuous variables were presented as mean (standard deviation) or median (Q1; Q3), whereas categorical variables were reported as absolute numbers and percentages.

To evaluate and compare the progression of surgical performance over time, the CUSUM method was applied, using operative time (skin-to-skin) as the primary performance indicator. The mean operative time across all procedures performed by all surgeons was used as the target outcome. For each case, the difference between the actual operative time and this target was calculated, and the deviations were cumulatively summed to construct the CUSUM learning curve for each surgeon.

The CUSUM score $C_{i}$ for the i-th operation (21):

$$C_{i}=\overset{n}{\underset{i=1}{\sum }}\;(X_{i}-\bar{X})$$

where $X_{i}$ represents the operative time of the i-th operation and $\bar{X}$ represents the mean operative time across all procedures of all surgeons.

To further assess each surgeon's progression, performance was compared across two defined phases: the Early Phase, comprising the first 10 operations performed by each surgeon, and the Late Phase, including all subsequent operations. For both phases, CUSUM curves were generated and analysed to identify reductions in operative time deviations and evidence of performance stabilization with increasing surgical experience.

Given that operative time may be influenced by patient and procedural complexity, a RA-CUSUM analysis was also performed. Instead of applying a fixed mean operative time to all cases, an expected operative time for each procedure was estimated using a multivariable linear regression model incorporating relevant predictors, including age, BMI, ASA score, previous abdominal surgery, and UICC stage. The difference between the observed and expected operative times was then cumulatively summed to generate individualized RA-CUSUM learning curves for each surgeon. This approach allows a more accurate and fair evaluation of surgical performance progression while accounting for variations in case complexity. All analyses were performed using the statistical software R (version 4.5.1).

### Ethics

The study adhered to the Declaration of Helsinki and received approval from the Ethics Committee of the Westfalen-Lippe Medical Association and the University of Münster (Ref. No. 2024-790-f-S). All patient data were anonymized prior to analysis to ensure confidentiality and compliance with institutional and national data protection regulations.

## Results

### Patient demographics

A total of 78 consecutive patients who underwent elective, RRC for histologically confirmed right-sided colon adenocarcinoma were screened for eligibility. Two cases requiring conversion to open surgery were excluded. Both conversions occurred during the initial phase due to severe obesity and unclear anatomy, rather than robotic system failure. Four additional cases using stapled intracorporeal anastomosis were excluded, resulting in a final cohort of 71 patients who underwent RRC with hand-sewn ICA.

A total of 71 patients were included, operated on by three colorectal surgeons who performed 27, 22, and 22 procedures, respectively. The cohort included 35 female (49.3%) and 36 males (50.7%) patients, with a mean age of 73.4 ± 9.8 years and a mean BMI of 26.5 ± 4.6 kg/m^2^ (Table 1). Most patients were classified as ASA II (38.0%) or ASA III (54.9%). Comorbidities were common: arterial hypertension (67.6%) and cardiac insufficiency (29.6%) were most frequent, followed by type 2 diabetes mellitus (19.7%) and coronary artery disease (15.5%). Prior abdominal surgery was present in 26.8% of patients, and 40.8% received anticoagulant therapy preoperatively.

**Table 1:** Baseline characteristics.

Characteristic	Surgeon 1	Surgeon 2	Surgeon 3	Overall
	(*n* = 27)	(*n* = 22)	(*n* = 22)	(*n* = 71)
Sex				
Female	16 (59.3%)	7 (31.8%)	12 (54.5%)	35 (49.3%)
Male	11 (40.7%)	15 (68.2%)	10 (45.5%)	36 (50.7%)
Age (Years)				
Mean (SD)	71.1 (11.9)	74.6 (9.36)	74.9 (6.77)	73.4 (9.77)
BMI (kg/m^2^)				
Mean (SD)	27.4 (5.09)	25.8 (3.45)	26.0 (5.06)	26.5 (4.63)
ASA-Score				
I	1 (3.7%)	0 (0%)	0 (0%)	1 (1.4%)
II	14 (51.9%)	5 (22.7%)	8 (36.4%)	27 (38.0%)
III	10 (37.0%)	16 (72.7%)	13 (59.1%)	39 (54.9%)
IV	2 (7.4%)	1 (4.5%)	1 (4.5%)	4 (5.6%)
Comorbidities				
Typ II diabetes mellitus	8 (29.6%)	3 (13.6%)	3 (13.6%)	14 (19.7%)
Coronary artery disease	2 (7.4%)	3 (13.6%)	6 (27.3%)	11 (15.5%)
Cardiac insufficiency	6 (22.2%)	9 (40.9%)	6 (27.3%)	21 (29.6%)
Arterial hypertension	17 (63.0%)	13 (59.1%)	18 (81.8%)	48 (67.6%)
Renal insufficiency	0 (0%)	1 (4.5%)	2 (9.1%)	3 (4.2%)
Chronic pulmonal disease (COPD)	2 (7.4%)	1 (4.5%)	2 (9.1%)	5 (7.0%)
Smoking	5 (18.5%)	6 (27.3%)	5 (22.7%)	16 (22.5%)
Previous abdominal surgery	9 (33.3%)	8 (36.4%)	2 (9.1%)	19 (26.8%)
Medical treatment				
Anticoagulant treatment	8 (29.6%)	11 (50.0%)	10 (45.5%)	29 (40.8%)

### Intra-operative outcomes

All procedures were completed robotically without intraoperative complications (Table 2). The median operative time was 165 min (Q1–Q3: 147–192), decreasing from 185 min for Surgeon 1 to 152 min for Surgeon 3, reflecting progressive efficiency with experience. Simultaneous resections were performed in 26.7% of patients, most commonly cholecystectomy (19.7%), followed by limited liver resections (4.2%) and salpingo-oophorectomy (2.8%).

**Table 2:** Operative outcomes.

Operative variable	Surgeon 1	Surgeon 2	Surgeon 3	Overall
	(*n* = 27)	(*n* = 22)	(*n* = 22)	(*n* = 71)
Operative time (min)^a^				
Median (Q1, Q3)	185 (162, 202)	166 (148, 195)	152 (141, 166)	165 (147, 192)
Simultaneous resections				
Cholecystectomy	8 (29.6%)	1 (4.5%)	5 (22.7%)	14 (19.7%)
Salpingo-oophorectomy	1 (3.7%)	0 (0%)	1 (4.5%)	2 (2.8%)
Atypic Liver resection	1 (3.7%)	0 (0%)	2 (9.1%)	3 (4.2%)
Intraoperative complications	0 (0%)	0 (0%)	0 (0%)	0 (0%)
Specimen extraction site				
Pfannenstiel incision	27 (100%)	22 (100%)	22 (100%)	71 (100%)
Using ICG	27 (100%)	22 (100%)	22 (100%)	71 (100%)
Preoperative bowel preparation (mechanical + antibiotics)	27 (100%)	22 (100%)	22 (100%)	71 (100%)

a Operative time = from skin incision to skin closure.

### Post-operative outcomes

Overall morbidity was 25.4%, including 9.9% major complications (Clavien–Dindo ≥ IIIb) (Table 3). The anastomotic leak rate was 5.6% (four cases), all of which were managed successfully by laparotomy and reconstruction with a new side-to-side isoperistaltic anastomosis. Other postoperative complications included bowel atony (12.7%), wound infection (2.8%), anastomotic bleeding (1.4%), and isolated cases of chyle leak, urinary tract infection, and pneumonia. The single episode of anastomotic bleeding (1.4%) was treated endoscopically. Cases of bowel atony were managed conservatively with nasogastric decompression, temporary cessation of enteral feeding, and administration of prokinetic agents. Reoperation was required in seven patients (9.9%), while 30-day mortality was zero. The median postoperative hospital stay was 8 days (IQR 7–12). Minor concomitant procedures, including limited liver wedge resections, were performed in a small number of patients and did not have a measurable impact on postoperative morbidity or overall operative outcomes.

**Table 3:** Post-operative outcomes.

Postoperative variable	Surgeon 1	Surgeon 2	Surgeon 3	Overall
	(*n* = 27)	(*n* = 22)	(*n* = 22)	(*n* = 71)
Post-operative complications				
Anastomotic leak	1 (3.7%)	2 (9.1%)	1 (4.5%)	4 (5.6%)
Anastomotic bleeding	1 (3.7%)	0 (0%)	0 (0%)	1 (1.4%)
Bowel atony	4 (14.8%)	4 (18.2%)	1 (4.5%)	9 (12.7%)
Chyle leakage	0 (0%)	0 (0%)	1 (4.5%)	1 (1.4%)
Pneumonia	0 (0%)	1 (4.5%)	0 (0%)	1 (1.4%)
Urinary tract infection	1 (3.7%)	0 (0%)	0 (0%)	1 (1.4%)
Wound infection	0 (0%)	1 (4.5%)	1 (4.5%)	2 (2.8%)
Intraperitoneal hematoma	0 (0%)	1 (4.5%)	0 (0%)	1 (1.4%)
Small bowel perforation	0 (0%)	1 (4.5%)	0 (0%)	1 (1.4%)
Clavien-Dindo < IIIb	5 (18.5%)	4 (18.2%)	2 (9.1%)	11 (15.5%)
Clavien-Dindo ≥ IIIb	1 (3.7%)	4 (18.2%)	2 (9.1%)	7 (9.9%)
Post-operative blood transfusion	2 (7.4%)	1 (4.5%)	0 (0%)	3 (4.2%)
30-day readmission	0 (0%)	0 (0%)	1 (4.5%)	1 (1.4%)
30-day reoperation	1 (3,7%)	4 (18,2%)	2 (9,1%)	7 (9,9%)
30-day mortality	0 (0%)	0 (0%)	0 (0%)	0 (0%)
Post-operative intensive care unit (days)				
Mean (SD)	0.556 (1.12)	0.818 (1.79)	0.364 (0.953)	0.577 (1.32)
Median (Q1, Q3)	0 (0, 1.00)	0 (0, 1.00)	0 (0, 0)	0 (0, 1.00)
Post-operative intermediate care unit (days)				
Mean (SD)	0.0741 (0.267)	0.455 (1.14)	0.0455 (0.213)	0.183 (0.683)
Median (Q1, Q3)	0 (0, 0)	0 (0, 0)	0 (0, 0)	0 (0, 0)
Post-operative length of hospital stay (days)				
Mean (SD)	10.5 (3.71)	10.3 (5.22)	8.77 (2.83)	9.90 (4.04)
Median (Q1, Q3)	9.00 (7.50, 13.0)	8.00 (7.00, 12.0)	8.00 (7.00, 11.5)	8.00 (7.00, 12.0)

### Histopathological findings

All resected tumors were adenocarcinomas (Table 4). The majority were staged as pT3 (49.3%), pN0 (63.4%), and UICC stage I–II (63.4%). Metastatic disease (cM1) was present at 14.1%, most frequently in the liver (7.0%) and less commonly in the lung (1.4%) or peritoneum (4.2%). R0 resection was achieved in 70 patients (98.6%). The only R1 resection occurred in a pT4 tumor with bladder infiltration, where the positive margin was confined to the bladder interface. The mean lymph-node yield was 29.6 ± 11.2, consistent across surgeons. An intact mesocolon (CME grade 0) was achieved in 90.1% of specimens.

**Table 4:** Histopathological outcomes.

Histopathological variable	Surgeon 1	Surgeon 2	Surgeon 3	Overall
	(*n* = 27)	(*n* = 22)	(*n* = 22)	(*n* = 71)
Type of the Tumor				
Adenocarcinoma	27 (100%)	22 (100%)	22 (100%)	71 (100%)
TNM pT-Stage				
pT1	5 (18.5%)	3 (13.6%)	3 (13.6%)	11 (15.5%)
pT2	6 (22.2%)	2 (9.1%)	6 (27.3%)	14 (19.7%)
pT3	13 (48.1%)	11 (50.0%)	11 (50.0%)	35 (49.3%)
pT4	3 (11.1%)	6 (27.3%)	2 (9.1%)	11 (15.5%)
TNM pN-Stage				
pN0	16 (59.3%)	13 (59.1%)	16 (72.7%)	45 (63.4%)
pN1	5 (18.5%)	3 (13.6%)	2 (9.1%)	10 (14.1%)
pN2	6 (22.2%)	6 (27.3%)	4 (18.2%)	16 (22.5%)
TNM pM-Stage				
M0	23 (85.2%)	18 (81.8%)	20 (90.9%)	61 (85.9%)
M1	3 (11.1%)	4 (18.2%)	3 (13.6%)	10 (14.1%)
Metastasis lung	0 (0%)	1 (4.5%)	0 (0%)	1 (1.4%)
Metastasis liver	2 (7.4%)	2 (9.1%)	2 (9.1%)	6 (8.4%)
Metastasis peritoneal	1 (3.7%)	1 (4.5%)	1 (4.5%)	3 (4.2%)
Resection status				
R0	27 (100%)	22 (100%)	21 (95.5%)	70 (98.6%)
R1	0 (0%)	0 (0%)	1 (4.5%)	1 (1.4%)
Tumor grading				
G1	2 (7.4%)	1 (4.5%)	2 (9.1%)	5 (7.0%)
G2	19 (70.4%)	20 (90.9%)	19 (86.4%)	58 (81.7%)
G3	6 (22.2%)	1 (4.5%)	1 (4.5%)	8 (11.3%)
UICC-Stage				
1	9 (33.3%)	5 (22.7%)	9 (40.9%)	23 (32.4%)
2	7 (25.9%)	8 (36.4%)	7 (31.8%)	22 (31.0%)
3	7 (25.9%)	5 (22.7%)	4 (18.2%)	16 (22.5%)
4	4 (14.8%)	4 (18.2%)	2 (9.1%)	10 (14.1%)
Quality of CME				
0	23 (85.2%)	19 (86.4%)	22 (100%)	64 (90.1%)
1	4 (14.8%)	3 (13.6%)	0 (0%)	7 (9.9%)
Retrieved lymph nodes				
Mean (SD)	32.1 (13.5)	27.7 (9.20)	28.4 (9.63)	29.6 (11.2)

### Learning curve analysis

**Figure 1:**
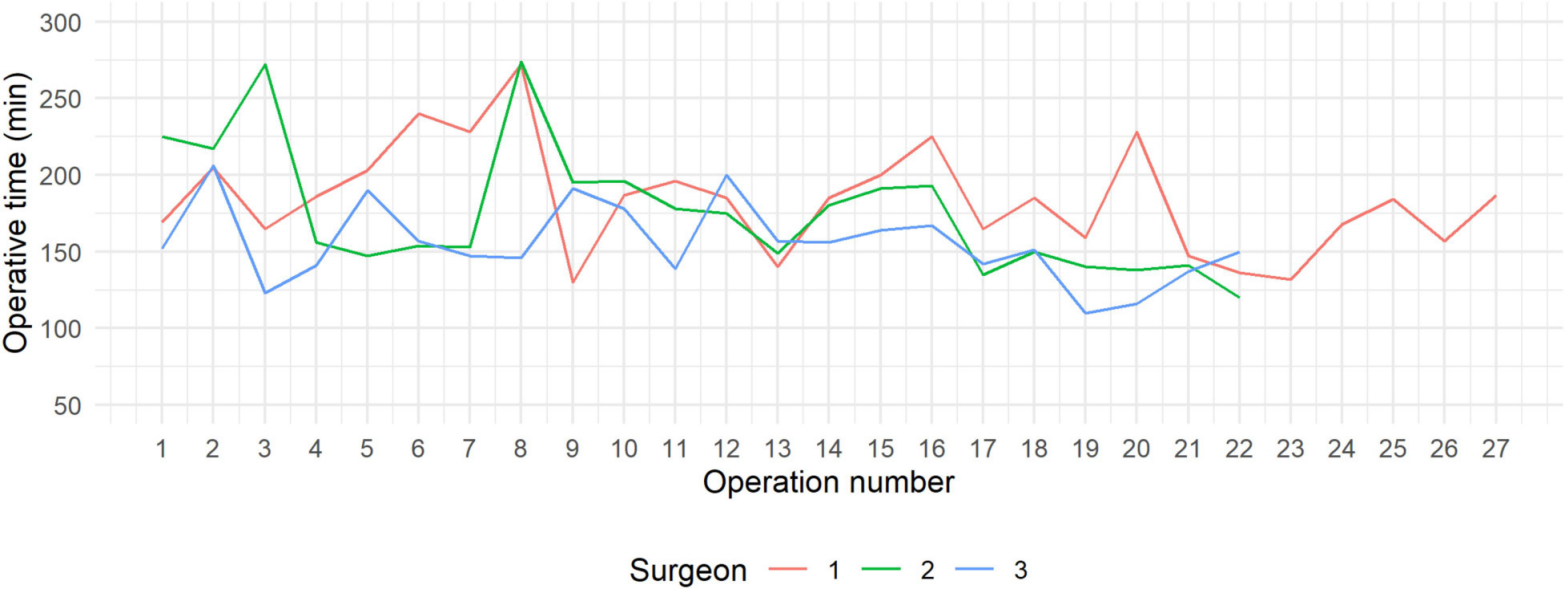
Operative time for each operation, categorized by surgeon.

The progression of operative proficiency was evaluated for each surgeon using CUSUM and RA-CUSUM analyses. Surgeons 2 and 3 each conducted 22 operations, whereas Surgeon 1 completed 27. Table 5 displays the average console time for the first 10 cases, as well as for cases beyond 10, along with the average overall operating time. Additionally, Figure 1 displays the individual learning curves of the respective surgeons. All three learning curves showed a similar pattern, with operative time decreasing as case numbers increased. Nevertheless, it is evident that Surgeon 3 typically has shorter operative times compared to the other two surgeons.

**Table 5:** Operative time by surgeon and learning phase (first 10 vs. subsequent procedures).

Operative time [Mean (SD)]	Surgeon 1	Surgeon 2	Surgeon 3
Overall	184 (34.8)	176 (41.8)	155 (25.5)
OP 1–10	199 (40.9)	199 (47.9)	163 (26.6)
OP ≥ 11	175 (28.5)	158 (24.5)	149 (23.7)

**Figure 2:**
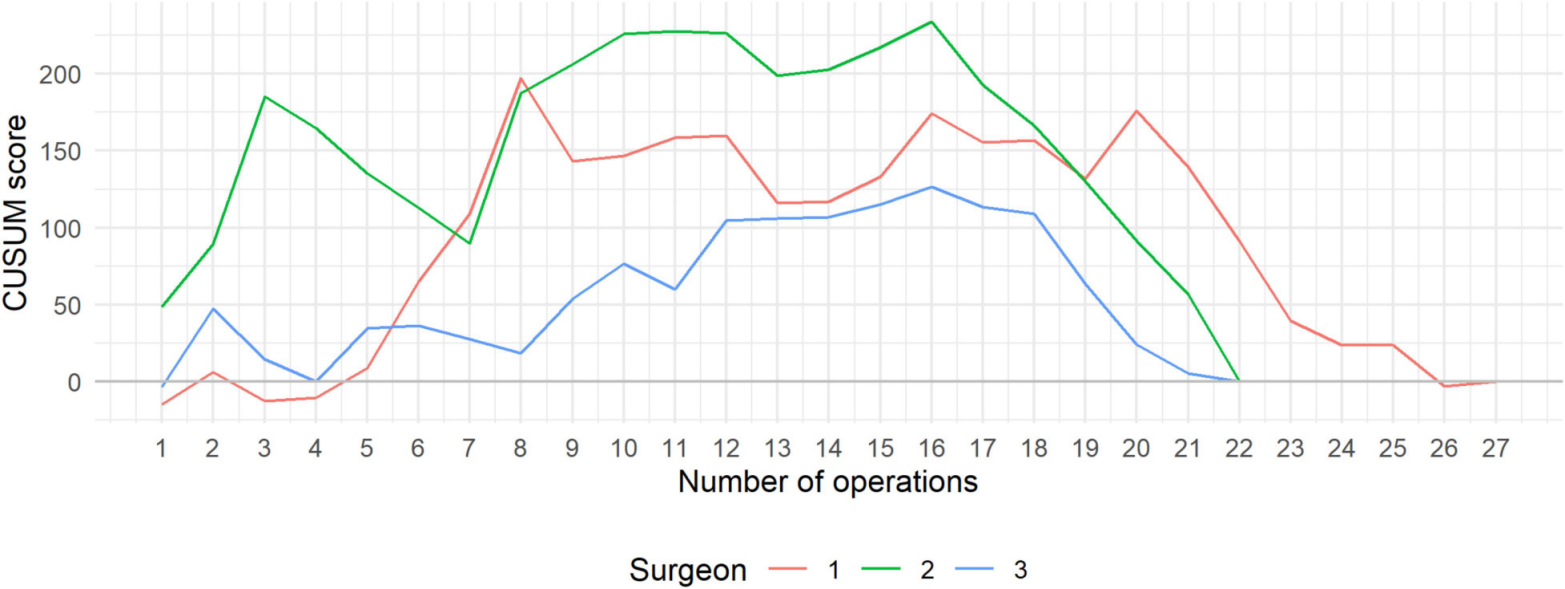
CUSUM learning curve categorized by surgeon.

Following the conversion of operative time into CUSUM scores, the CUSUM learning curves for all three surgeons are illustrated in Figure 2. The CUSUM curve associated with Surgeon 3 revealed a lower slope in comparison to the other two surgeons. However, all three CUSUM curves maintained a similar overall trend: an initial upward phase representing the learning and adaptation period, followed by a turning point near the 16th operation. This was subsequently followed by a decline, indicating enhanced efficiency and better operative performance.

**Figure 3:**
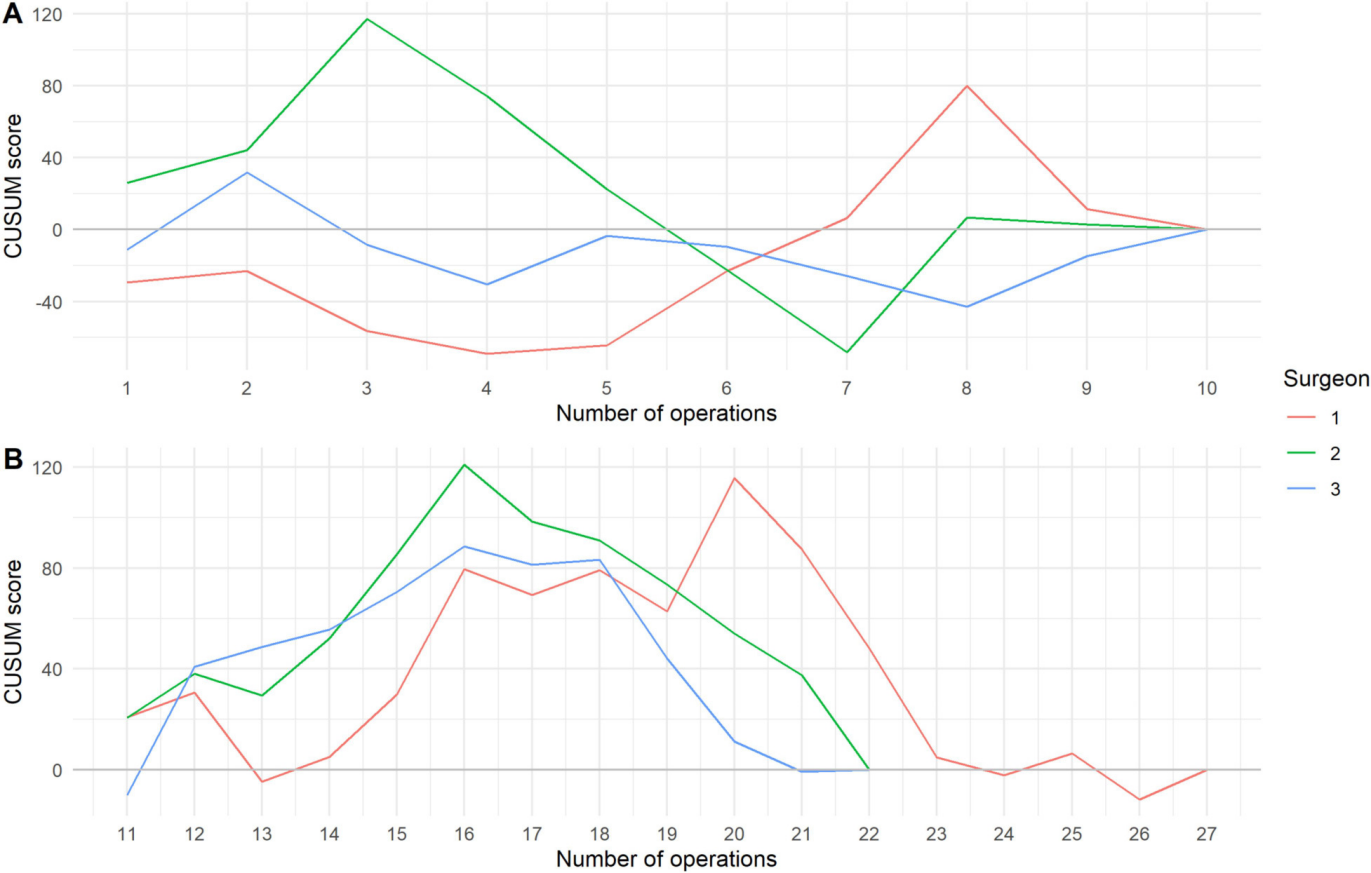
CUSUM learning curve classified by surgeon and the total number of operations conducted. **(A)** operations 1–10 and **(B)** operations >10.

Figure 3 illustrates the segmented CUSUM curves for each surgeon, divided into two operational phases: **(A)** operations 1–10, representing the initial learning and adaptation period, and **(B)** operations >10, representing the consolidation or proficiency phase.

During the early phase (A), all surgeons exhibited fluctuating CUSUM scores, indicating varying performance and longer operative times typical of the initial learning stage. Surgeon 2 showed the steepest early rise, reflecting a slower adaptation period, whereas Surgeon 1 reached performance stabilization earlier. In the later phase (B), all curves demonstrated a consistent decline and eventual plateau, signifying progressive efficiency and technical consistency. The transition between phases occurred around the 15th–17th case, which corresponds closely to the inflection points identified in Figure 2, confirming that the learning curve plateau was achieved after approximately 15–20 procedures per surgeon.

**Figure 4:**
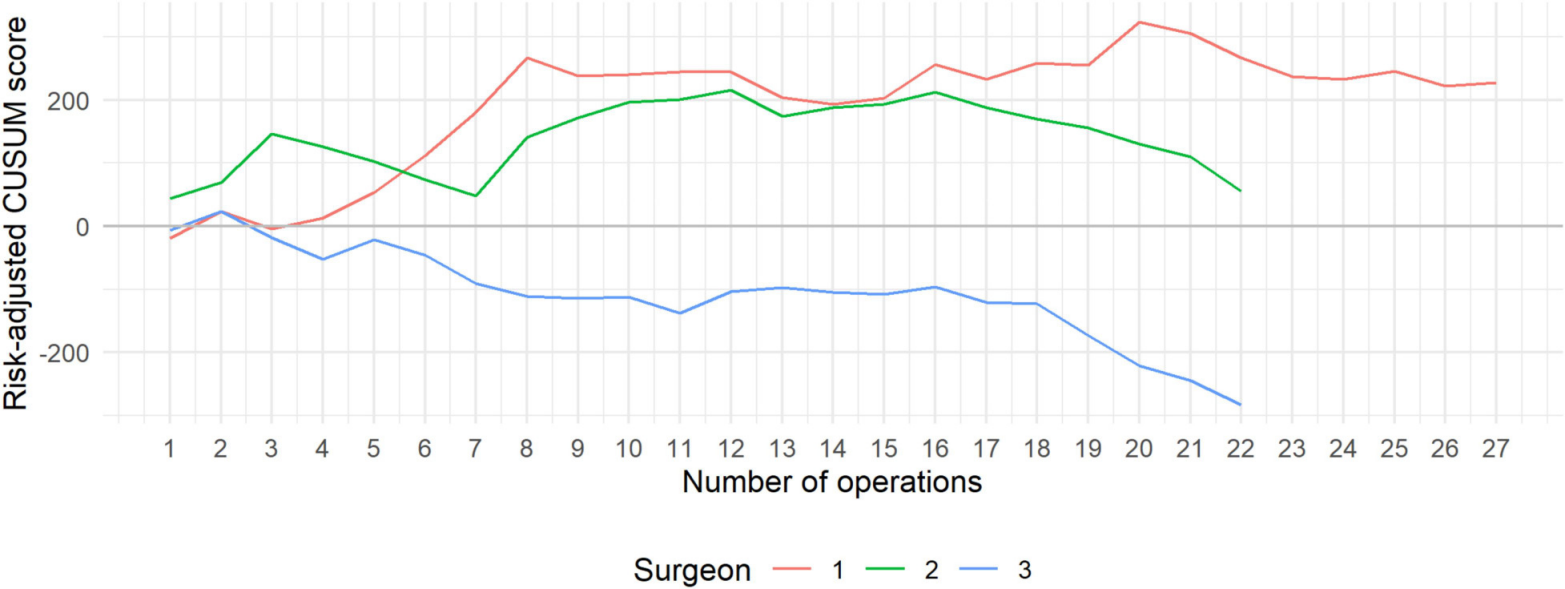
Risk-adjusted CUSUM learning curve classified by surgeon and the total number of operations conducted.

Figure 4 displays the risk-adjusted CUSUM scores. The curves indicate that Surgeons 1 and 2 consistently recorded operative times longer than expected, even after adjusting for variations in case complexity. In contrast, Surgeon 3 demonstrated shorter-than-expected operative times, with a clear downward trend that reflects increasing efficiency over time.

## Discussion

This study demonstrates that RRC with CME, CVL, and hand-sewn ICA can be safely implemented within a multi-surgeon robotic program, achieving low morbidity, satisfactory oncological outcomes, and a defined learning phase after approximately 15–20 procedures per surgeon. These results are consistent with previously published literature confirming the feasibility and oncological adequacy of robotic CME techniques while contributing novel data on the learning process for hand-sewn ICA in a real-world institutional setting (2, 5, 22).

### Operative and oncologic outcomes

The median operative time of 165 min was shorter than the 180–330 min range reported in previous systematic reviews, demonstrating procedural efficiency despite the multi-surgeon setting and the use of a hand-sewn intracorporeal anastomosis. The overall complication rate was within the 20%–30% (1, 4) range reported in recent studies, while major complications (Clavien–Dindo ≥ IIIb) occurred in 9.9%, consistent with the published range of 2%–11.5% (1, 4). Most complications were minor and managed conservatively, and no mortality occurred, reflecting the safety profile of robotic CME.

The anastomotic leak rate in this series was 5.6%, slightly higher than values reported in previous RRC studies. Reported leak rates for hand-sewn intracorporeal anastomoses range between 2.9% and 3.8%, while systematic reviews have described an overall range of 0%–2%, predominantly for stapled techniques (1, 7, 14). All four leaks occurred during the early learning phase, within the first 10 cases of each surgeons—two in one surgeon and one in each of the other two—reflecting both the small sample size and the expected early adaptation period associated with the exclusive use of a hand-sewn technique. Importantly, no further leaks were observed once proficiency was achieved, underscoring the influence of operative experience on anastomotic outcomes.

The median postoperative stay of 8 days was longer than in some international reports but is consistent with German DRG-guided care pathways, which typically involve prolonged inpatient monitoring following colorectal surgery. Notably, ICU and IMC stays were short, and no mortality occurred.

The mean lymph-node yield of 29.6 ± 11.2 and R0 resection rate of 98.6% confirm oncologic adequacy and reflect adherence to CME principles. Previous reviews have shown that robotic CME achieves equivalent or superior specimen quality compared with laparoscopic CME, with higher rates of intact mesocolic fascia and consistent central vessel ligation (1, 4, 23, 24). Moreover, the improved visualization and dexterity provided by the robotic platform likely contribute to the low conversion rate and precise vascular dissection observed in our study. Beyond oncologic adequacy, an important technical aspect of RRC concerns the optimal method of intracorporeal anastomosis.

### Hand-sewn intracorporeal anastomosis technique

A distinctive feature of this series is the consistent use of a hand-sewn ICA technique. Evidence comparing hand-sewn and stapled ICA remains limited (25). A multicenter prospective study demonstrated comparable leak rates and morbidity between stapled and hand-sewn ICAs during robotic right colectomy, emphasizing that both techniques are safe when performed by experienced teams (7).

Recent evidence (26, 27) supports the intracorporeal anastomotic approach—regardless of technique—as it is associated with shorter hospital stays, fewer wound infections, and a lower incidence of incisional hernias compared with extracorporeal anastomosis. The choice between stapled and hand-sewn reconstruction should be guided by surgeon expertise and familiarity rather than concerns regarding safety or oncologic adequacy.

Notably, robotic systems facilitate intracorporeal suturing through enhanced instrument dexterity and stable three-dimensional visualization, thereby reducing the technical gap between stapled and hand-sewn approaches. Moreover, the robotic platform expands the feasibility of manual intracorporeal anastomosis, allowing for precise perfusion assessment and the creation of tension-free, well-aligned anastomoses (28).

### Learning curve interpretation

Quantitative learning-curve analysis using CUSUM and RA-CUSUM provides valuable insight into the adoption trajectory of robotic colorectal procedures. In this series, the inflection point at approximately the 16th case marked the transition from the learning to the proficiency phase, consistent with prior reports suggesting 25–35 cases for RRC (2, 16).

The use of RA-CUSUM adds robustness by adjusting patient- and case-related factors such as BMI, ASA class, and tumor stage, thereby distinguishing performance improvement from favorable case selection. All three surgeons demonstrated progressive reduction in operative time variability and convergence towards consistent efficiency. Prior minimally invasive experience likely contributed to the early plateau observed in the CUSUM curves. The surgeons' established proficiency in advanced laparoscopic and robotic colorectal procedures may explain the relatively rapid transition to stable operative performance.

This observation aligns with previous reports indicating that prior laparoscopic experience can significantly shorten the robotic learning curve, consistent with our institutional findings (29).

## Limitations

This study has several limitations. Its retrospective, single-center, design and moderate sample size limits the generalizability of the results. Nevertheless, all data were prospectively recorded, and procedural standardization helped minimize selection and performance bias. The inclusion of three surgeons enhances external validity but introduces a degree of inter-operator variability that may have influenced operative times and learning-curve trajectories. Prospective multicenter validation is warranted to confirm the learning-curve thresholds identified for robotic CME with hand-sewn ICA and to evaluate long-term oncologic outcomes.

Learning curve results should also be interpreted in the context of each surgeon's prior laparoscopic and robotic experience, which likely facilitated earlier proficiency.

Despite these limitations, the consistency of perioperative and oncologic outcomes across three surgeons demonstrates the feasibility and reproducibility of adopting a standardized robotic CME technique within a single institutional framework. Structured implementation—including proctoring, uniform port configuration, and adherence to the SMV-first approach—likely contributed to the uniformity and safety of results.

## Conclusions

RRC with CME, CVL, and hand-sewn ICA is a safe, feasible, and oncologically sound technique. The learning curve can be completed after approximately 15–20 procedures per surgeon. These findings support integrating hand-sewn ICA into structured robotic colorectal training programs.

## Data Availability

The original contributions presented in the study are included in the article/Supplementary Material, further inquiries can be directed to the corresponding author.
