# Supplementary material for: Robotic right colectomy with complete mesocolic excision, central vascular ligation and hand-sewn intracorporeal anastomosis: feasibility, safety, and learning curve analysis
Source: Front Surg. 2026 Jan 14;12:1740276. doi: 10.3389/fsurg.2025.1740276 (PMC12847402; doi:10.3389/fsurg.2025.1740276)
Supplement: Supplementary file 1 [file Supplementaryfile1.docx]

**Supplementary Material to**

**Robotic Right Hemicolectomy with Complete Mesocolic Excision, Central Vascular Ligation, and Hand-Sewn Intracorporeal Anastomosis: Feasibility, Safety, and Learning Curve Analysis**

Zsolt Madarasz, Krysztof Nowakowski, Michael Leitz, Bogdan-Cornel Sturzu, Anas Baltamar, Kira Baginski, Annika Hoyer, Jens Hoeppner, Fabian Nimczewski, Miljana Vladimirov


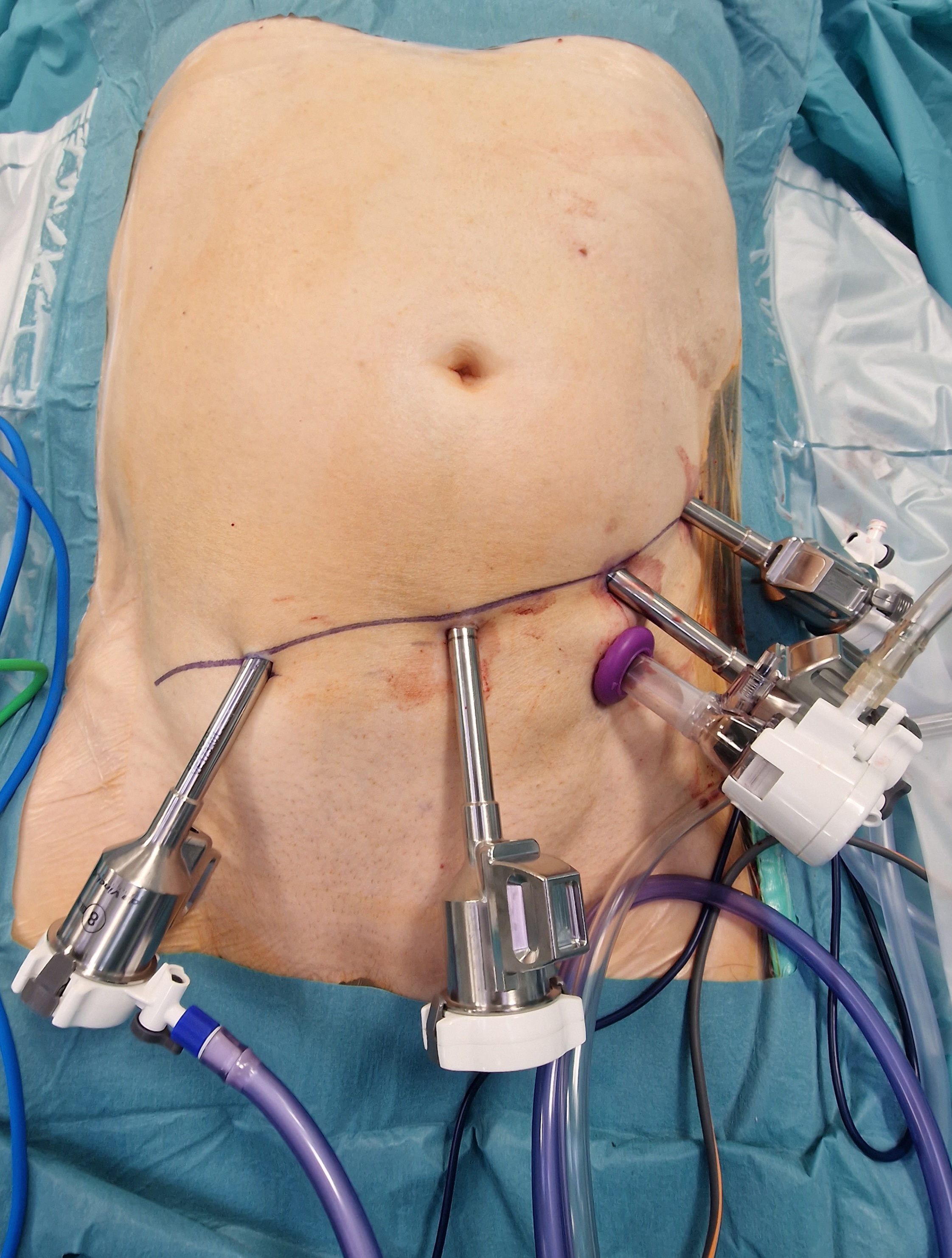


Figure S1 - **Oblique Trocar-Placement**


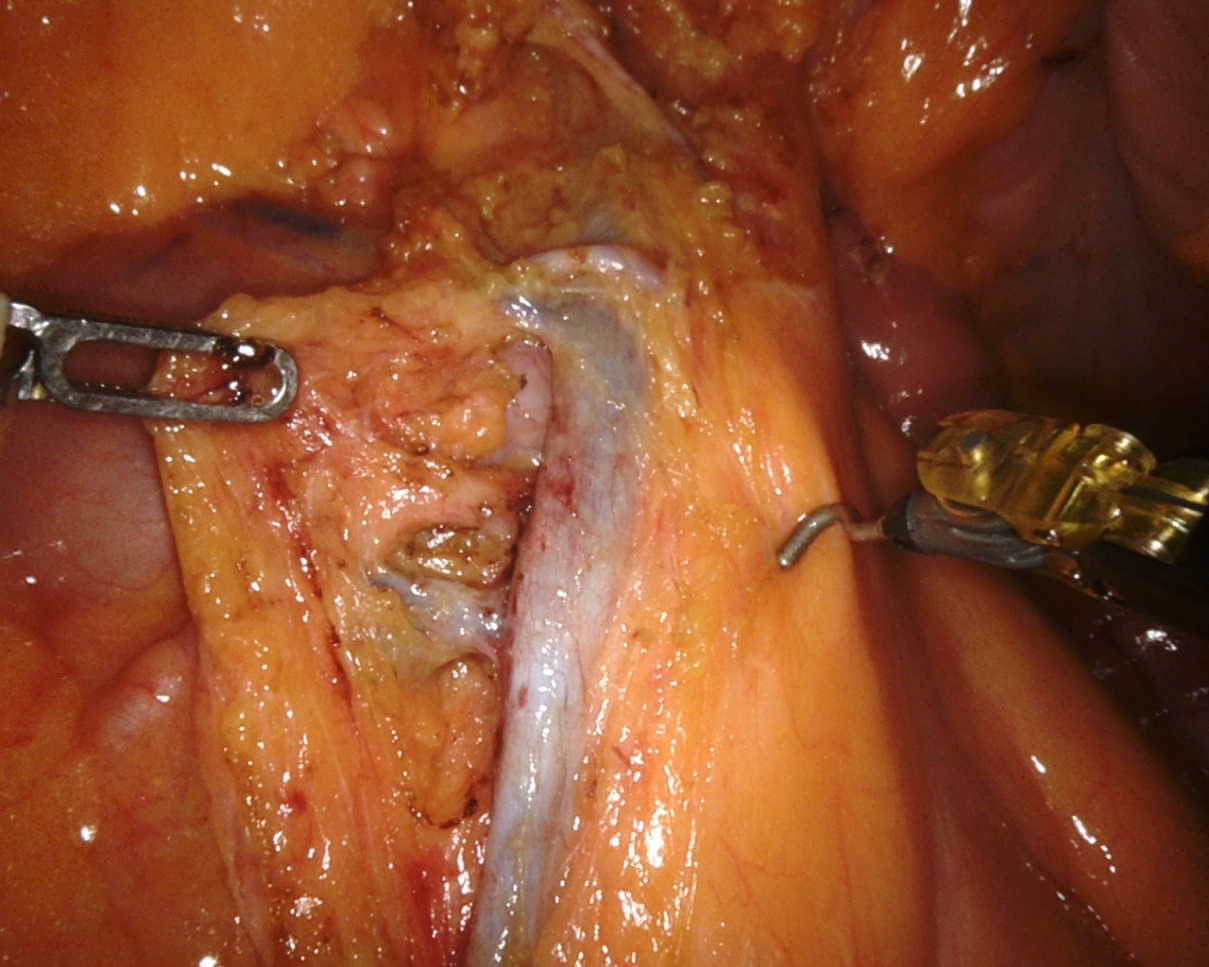


Figure S2 - **Complete Mesocolic Excision (CME) with Central Vascular Ligation (CVL)**


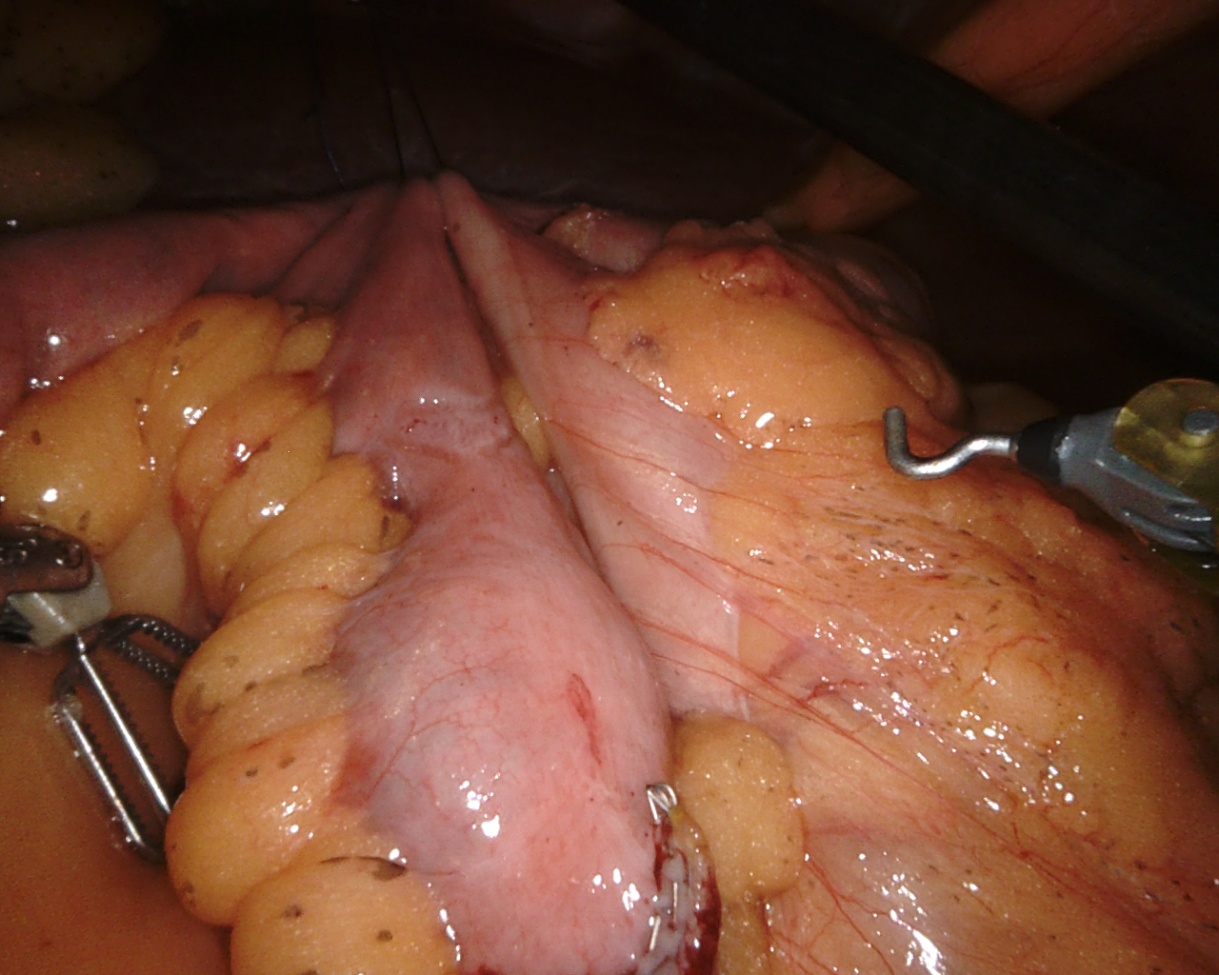


Figure S3 - **Hand-sewn Anastomosis Step 1**


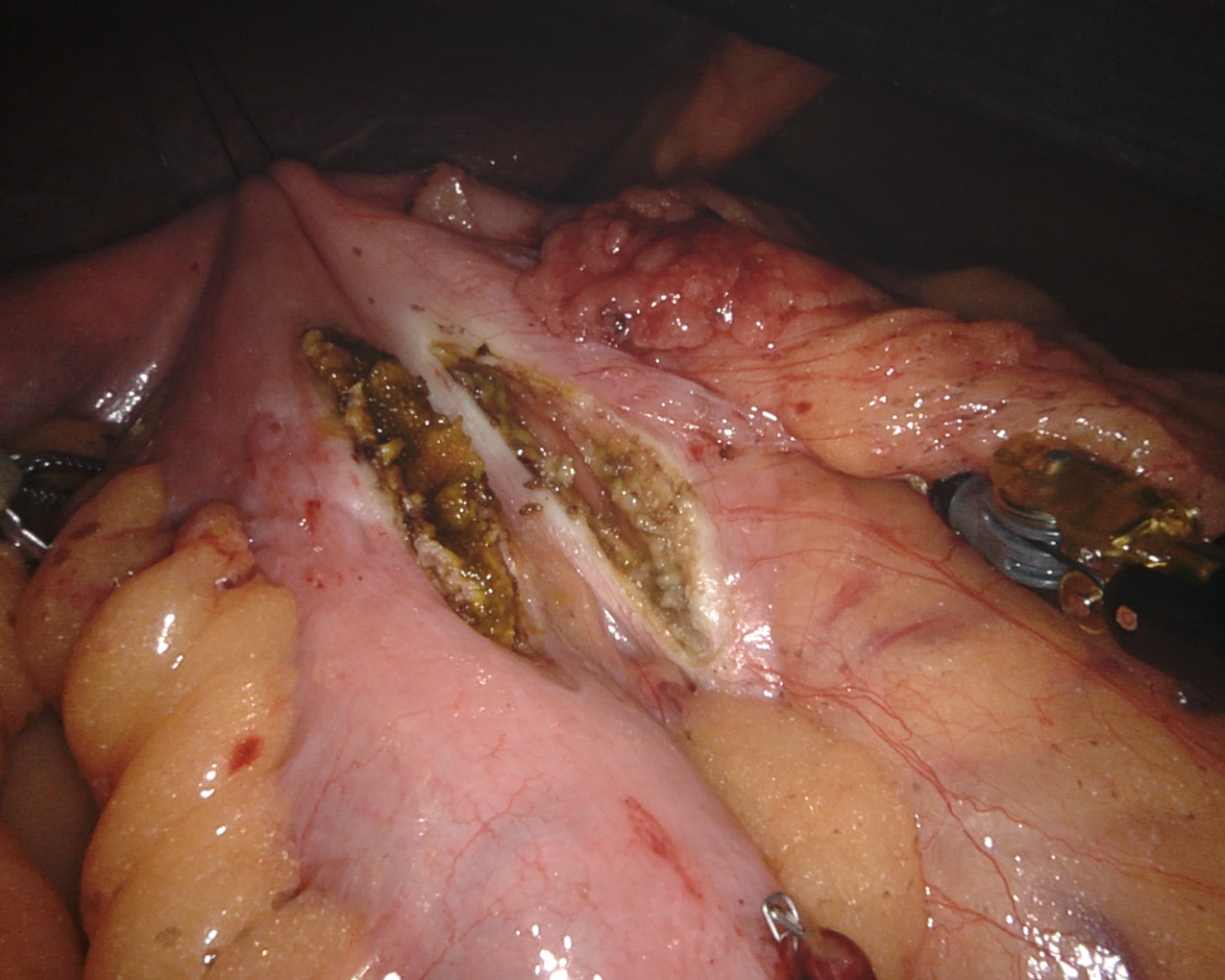


Figure S4 - **Hand-sewn Anastomosis Step 2**


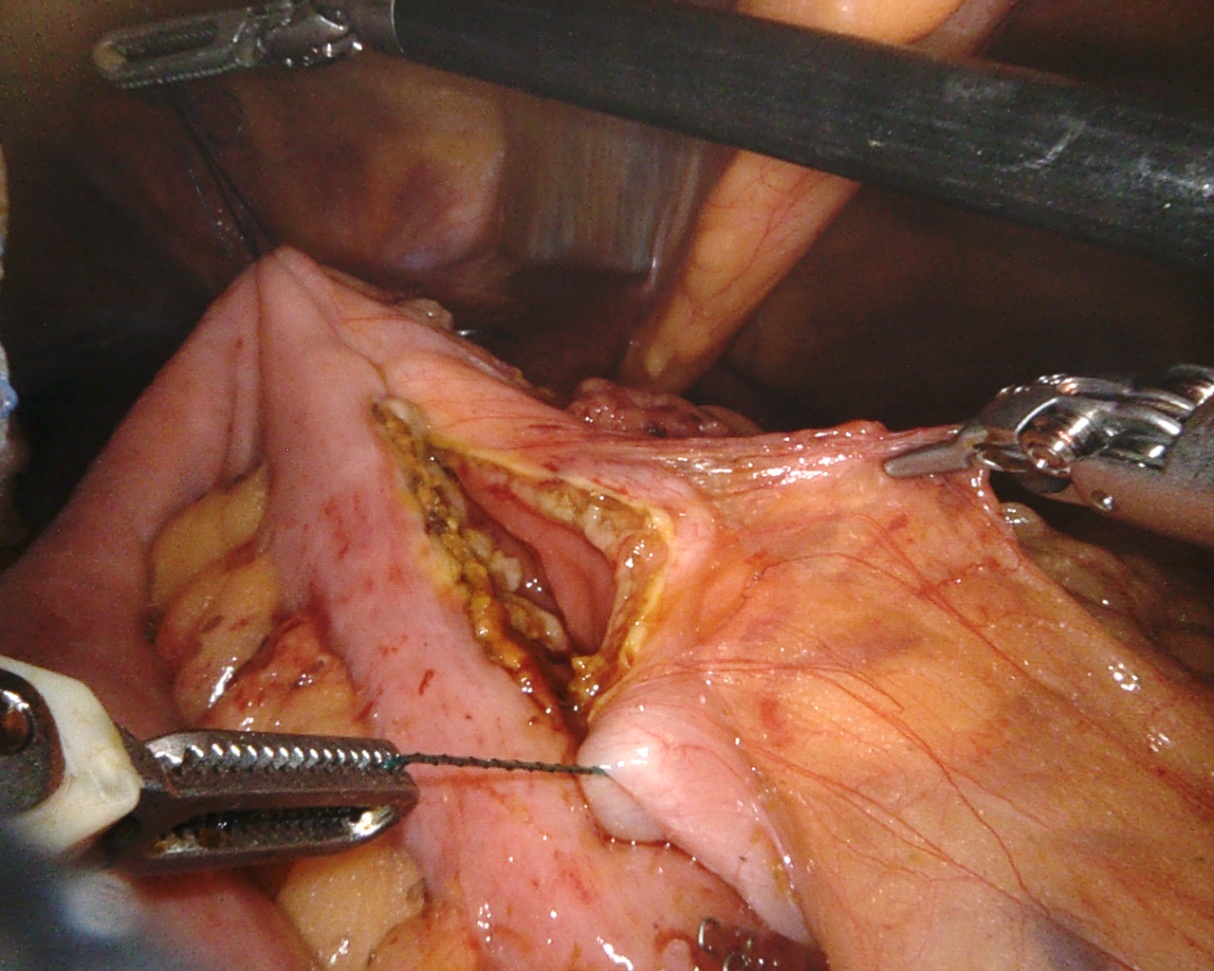


Figure S5 - **Hand-sewn Anastomosis Step 3**


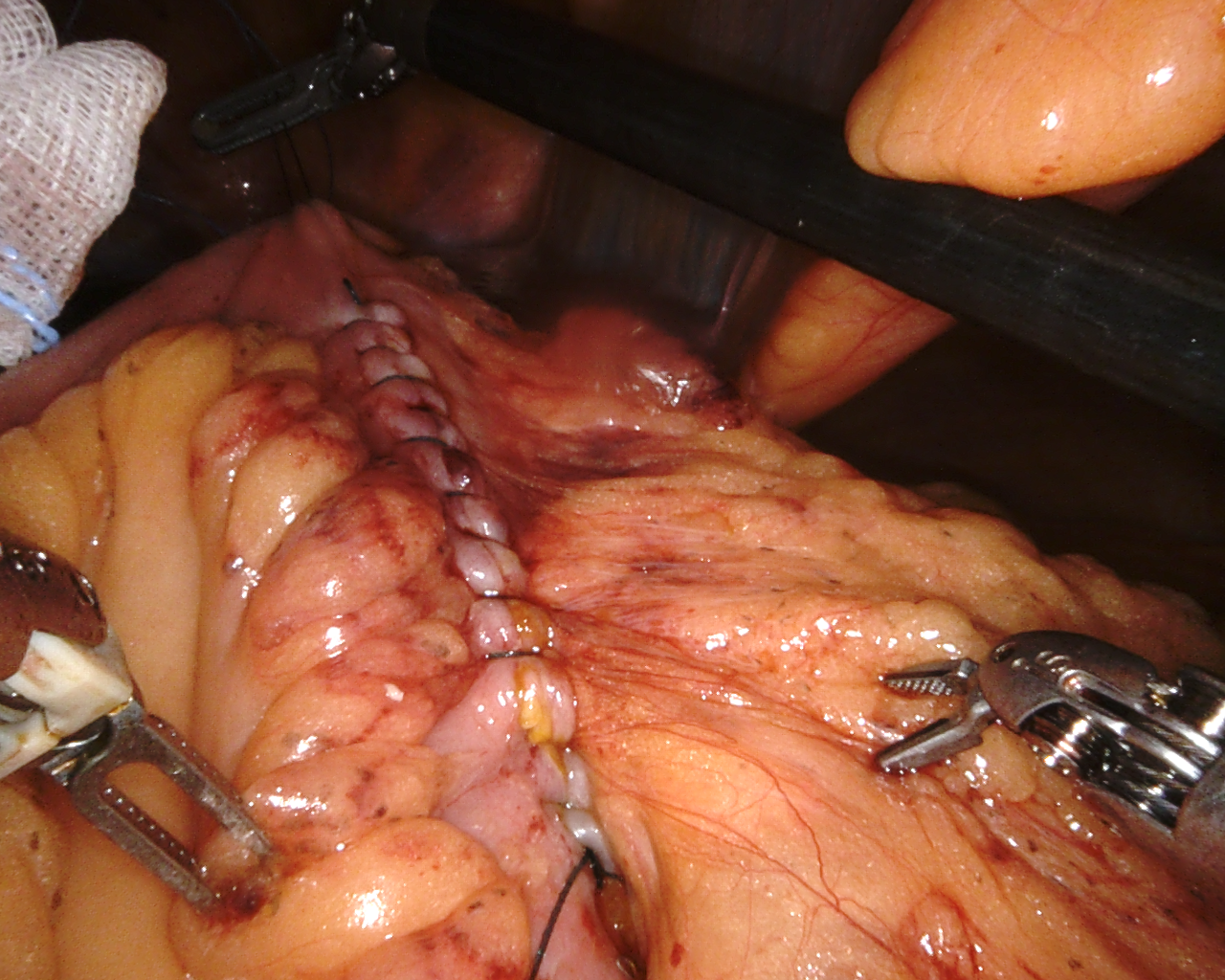


Figure S6 - **Hand-sewn** **Anastomosis Step 4**
